# The Chemistry of the Table

**Published:** 1893

**Authors:** 


### THE CHEMISTRY OF THE TABLE.

Though our foods are so varied, the different kinds of feeding materials or "food stuffs" they contain are few, or at least for practical purposes may be reckoned under a few heads. Thus food stuffs are : 1. Albuminoids and other substances containing nitrogen. 2. Fat, starch and sugar. 3. Mineral substances, chiefly common salt and phosphates. 4. Water. 5. Food adjuncts.

The principal function of the above classes of food-stuffs are as follows : 1. Albuminoids, etc., are oxidized by the air which is inhaled into the body, and go to form the muscle and flesh. The process of oxidation gives out heat, and hence these flesh forming foods assist also to keep up the heat of the body. 2. Fat, starch and sugar are oxidized in the body, thereby acting as heat givers, but they do not form muscle and flesh. In hard bodily work it is necessary to increase the supply of heat giving food.

The waste of muscle and flesh does not increase with increased bodily labor in nearly the same proportion as does the demand upon the heat of the body. Fat has about two and one-third times the warming power of starch and sugar, there being a larger proportion of carbon and hydrogen to be oxidized. Hence fat is the principal food stuff to be added to the diet to meet the demand of extra bodily work or the corresponding tax of a colder climate. Fat can be stored up in the body (as a layer under the skin), where it acts as a store of heat giving material, which can be drawn upon as required by the system. 3. Of the mineral substances taken into the body the lime salts and phosphates form the solid fabric of the bones. 4. Water constitutes about two-thirds by weight, of the substances of the body.

The water taken in as food is required both as a constituent of the body, and also as a carrier of food in and through the system.

5. Food adjuncts are of importance more on account of their effects (whether stimulating or sedative) upon the nervous system, and on account of their effect on the palate, than for any actual power of nourishing or sustaining the fabric of the body. The most important are alcohol and the alkaloids contained in tea, coffee and cacao. The daily ration of an adult under ordinary conditions, according to Professor Church, should contain the different food stuffs in about the following quantities :

| | Oz. avoirdupois. |
|---|---|
| 1. Water | 88.66 |
| 2. Albuminoids | 4.25 |
| 3. Starch and sugar | 11.40 |
| 4. Fat | 3.77 |
| 5. Mineral food | 1.03 |
| | 109.11 |

The small quantity of food adjuncts is not included in the above table. The actual weight of food eaten will exceed the above total by about one ounce on account of fibrous material, either vegetable or animal, which is taken with the food, but which is not assimilated by the body. The food stuffs three and four have the same function, viz., that of keeping up the heat of the body. Weight for weight, fat has about two and one-third times the heating power of starch. Of course it is not only the quantity of the food stuffs which has to be taken into account in judging whether the diet is a suitable one or not; we must also take account of the proportion between the amounts of the different food stuffs. The most important ratio, the actual value of which should, however, depend upon the amount of bodily work done, is the ratio of flesh formers to heat givers. If the fat be calculated according to its "starch equivalent," the ratio should be about 1.4¾. The ratio of flesh formers to heat givers is termed the nutrient ratio, and a table of the nutrient ratio of different classes of food is a valuable guide in the determination of a diet.

The most important flesh formers are the lean of meat and green vegetables. The starchy foods, as rice and potatoes, are, generally speaking, cheap and easily obtained. The fat of meat supplies the heat givers in a more concentrated form, though not so cheaply. The form in which the various food stuffs may be taken must depend largely upon the mode of life. Thus, a laboring man who works in the fields may make his dinner off a dish of cabbage and fat bacon, deriving the flesh formers from the vegetable, and the heat givers from the animal food. For men of sedentary habits such a combination would be scarcely suitable. A lean chop with potatoes—a very usual luncheon for a business man—supplies the same food stuffs in a lighter form. In this case the flesh formers are derived from the animal food, and the heat givers from the vegetable.

